# The presumed MTH1-inhibitor TH588 sensitizes colorectal carcinoma cells to ionizing radiation in hypoxia

**DOI:** 10.1186/s12885-018-5095-x

**Published:** 2018-11-29

**Authors:** Mosche Pompsch, Julia Vogel, Fabian Classen, Philip Kranz, George Iliakis, Helena Riffkin, Ulf Brockmeier, Eric Metzen

**Affiliations:** 1 Institut für Physiologie, Universität Duisburg-Essen, Hufelandstraße 55, D45122 Essen, Germany; 2 Institut für Medizinische Strahlenbiologie, Universität Duisburg-Essen, Hufelandstraße 55, D45122 Essen, Germany

**Keywords:** 8-oxo-Guanosin, DNA damage repair, MutT homologue-1, Oxygen

## Abstract

**Background:**

The nudix family member enzyme MutT homologue-1 (MTH1) hydrolyses the oxidized nucleotides 8-oxo-dGTP and 2-hydroxy-dATP and thus prevents the incorporation of damaged nucleotides into nuclear and mitochondrial DNA. Therefore MTH1 was proposed to protect cancer cells from oxidative DNA lesions and subsequent cell death. We investigated whether the bona fide MTH1 inhibitor TH588 affects responses of cultured colorectal tumor cells to ionizing radiation (IR) in normoxia and in moderate or severe hypoxia.

**Methods:**

TH588 was tested in cell viability and survival assays (tetrazolium dye (MTT), propidium iodide staining, caspase-3 activity, and colony formation assays (CFA)) in colorectal carcinoma cells (HCT116 and SW480) in combination with IR in normoxia and in hypoxia. Additionally, MTH1 was targeted by lentiviral shRNA expression. Human umbilical vein endothelial cells (HUVEC) were assessed in MTT assays.

**Results:**

In all cell lines tested, TH588 dose-dependently impaired cell survival. In CFAs, TH588 and IR effects on carcinoma cells were additive in normoxia and in hypoxia. Using 3 different shRNAs, the lentiviral approach was detrimental to SW480, but not to HCT116.

**Conclusions:**

TH588 has cytotoxic effects on transformed and untransformed cells and synergizes with IR in normoxia and in hypoxia. TH588 toxicity is not fully explained by MTH1 inhibition as HCT116 were unaffected by lentiviral suppression of MTH1 expression. TH588 should be explored further because it has radiosensitizing effects in hypoxia.

## Background

MutT Homologue-1 (MTH1) has been in the focus of biomedical and cancer research recently [1–3]. The mammalian enzyme MTH1 is the product of the NUDT1 gene and detoxifies the oxidized nucleotides 8-oxo-dGTP and, to a lesser extent, 2-OH-dATP. By hydrolysis of 8-oxo-dGTP, MTH1 prevents incorporation of 8oxoG into DNA [4]. Consequently, targeting this enzymatic function has been proposed to induce single strand breaks and G:C to T:A transversion mutations during DNA replication [5]. The MTH1 inhibitor TH588 was identified by Gad and co-authors in 2014 [6] and has been used in several studies subsequently [7–9]. Other investigators have generated inhibitors independently as reviewed very recently [10]. Interestingly, crizotinib, a drug which is in clinical use and regarded as a tyrosin kinase inhibitor, has also been reported to inhibit MTH1 [11, 12]. These compounds including TH588 bind to the active site of MTH1 and thus prevent access of 8-oxo-dGTP. The halfmaximal inhibitory concentration (IC50) of TH588 has been reported to be approximately 5 nM in enzyme activity assays while low micromolar concentrations were required to inhibit growth in cell culture experiments [6]. Remarkably, in the same publication toxicity is proposed to be limited to tumor cells as VH10 fibroblasts that were suggested to represent untransformed cells were virtually unaffected by TH588 thus inferring that MTH1 inhibitors would act on tumor cells selectively if used in vivo.

However, this concept has been challenged very recently. A series of efficient MTH1 inhibitors have been reported not to affect viability of cultured tumor cells [13] while TH588 reduced cell viability in the same study. Another group of authors identified tubulin as the main intracellular target of TH588 [14], which is an effect similar to well-established chemotherapeutic agents such as vinca alkaloids and taxanes.

In an effort to explain these controversial results we tested TH588 in two different carcinoma cell lines. We chose colorectal carcinoma because this is one of the most frequent tumor entities. Secondly, our intention was to test TH588 in combination with ionizing radiation (IR) which is frequently used in colorectal carcinoma patients. Of particular importance, one very recent study has suggested “radiosensitizing” activity of TH588 in neuroendocrine tumor cells [7]. IR is known to cause single and double strand breaks of the DNA at least in part via generation of reactive oxygen species (ROS). Therefore, it is indeed plausible that IR and TH588 inhibition which allows incorporation of oxidized nucleotides such as 8oxoG into DNA act synergistically. Of particular interest in this context is the question whether TH588 also affects cell viability in hypoxia. A lack of oxygen severely limits the efficiency of IR which has led to the definition of the oxygen enhancement ratio: most tumor cells are approximately 2.5 times more sensitive to IR in normoxia as compared to hypoxia. This also translates to a clinical setting where hypoxic areas of the tumor are frequently radioresistant and thus contribute to a poor treatment outcome of radiotherapy [15]. To define whether a “radiosensitizing” effect is detectable in colon carcinoma cells we therefore combined IR with TH588 in normoxia as well as in moderate (1% O_2_) and severe hypoxia (0.1% O_2_).

## Material and methods

### Reagents

TH588 was provided by Thomas Helleday (Karolinska Institutet, Stockholm, Sweden). Etoposide, doxycycline, Ac-Asp-Glu-Val-Asp-7-Amino-4-methylcoumarin (Ac-DEVD-AMC), 3-(4,5-dimethylthiazol-2-yl)-2,5-diphenyltetrazolium bromide (MTT), dimethylsulfoxide (DMSO), puromycin, polybrene, propidium iodide (PI) and Hoechst33342 were purchased from Sigma (Munich, Germany).

### Cell culture, transfection and lentiviral transduction

HEK293T cells (ACC635, DSMZ, Braunschweig, Germany) were cultured in high-glucose DMEM (Invitrogen, Darmstadt, Germany). HCT116 cells (ATCC-CCL-247, LGC Standards, Wesel, Germany) were cultured in McCoy’s 5A (Lonza, Basel, Switzerland). SW480 cells (ATCC-CCL-228, hairpin C shRNA-MTH1 transduced cells provided by T. Helleday and U. Warpman Berglund, Karolinska Institutet, Stockholm, Sweden) were cultured in RPMI1640 (Life Technologies, Carlsbad, CA, USA). Human umbilical vein endothelial cells (HUVECs, PromoCell, Heidelberg, Germany) were cultured in Endothelial Cell Growth Medium (PromoCell). Media were supplemented with 10% fetal bovine serum and penicillin/streptomycin. All cell lines were tested negative for mycoplasma contamination by PCR on a monthly basis. For transient transfection GeneJuice (Novagen/Merck Chemicals, Darmstadt, Germany) was used in a 3: 1 ratio (μl reagent/μg DNA). Lentiviral particles were produced in HEK293T cells as described previously [16]. The non-targeting tetracyclin-inducible pLKO-Tet-on-shRNA-NT contained the sequence 5’-CAACAAGATGAAGAGCACCAA-3′. The pLKO-Tet-on-shRNA-MTH1 contained the sequence 5’-CCTGAGCTCATGGACGTGCAT-3′ for MTH1 mRNA hairpin A and 5’-CCTGCTTCAGAAGAAGAAATT-3′ for MTH1 mRNA hairpin B. For transduction 2 × 10^5^ cells were incubated with 2 × 10^5^ transduction units for 16–24 h in the presence of 8 μg/ml polybrene. Cells were selected by treatment with 2 μg/ml puromycin for 5–7 d. Gene knockdown was induced by addition of 250 ng/ml doxycycline. Cells were irradiated using an X-ray machine (X-rad 320, PXI) operated at 320 kV, 12.5 mA with a 1.65 mm aluminum filter (dose rate: 3.6 Gy/min).

### Viability

MTT assays were used to quantify cell viability. 2 × 10^3^ HCT116 or 3 × 10^3^ SW480 cells were plated into 96-well plates, treated and incubated for 72 h. Either 8 × 10^3^ (exponentially growing) or 40 × 10^3^ (approximately 90% confluent) HUVECs were tested in the same way. After incubation with MTT for 4 h at 37 °C the cells were lysed. Absorbance was measured at 540 nm (Synergy HT, BioTek).

### Fluorescence microscopy

To quantify cell death, 1 × 10^5^ HCT116 cells were plated into 6-well plates. After 12 h the cells were irradiated. Immediately following IR reagents were applied. Then the cells were incubated for another 48 h. Cells were stained with 2.5 μg/ml PI which indicates cell death and with 0.85 μg/ml Hoechst33342. Fluorescence microscopy (Zeiss Axiovert 200 M, Carl Zeiss, Oberkochen, Germany) was used to determine the quotient of dead cells (PI) divided by total cells (Hoechst 33342).

### Colony formation assay

CFAs were performed to measure long term survival as reported recently [17]. In brief, HCT116 or SW480 cells were plated into rat collagen type I (R&D Systems, Wiesbaden, Germany) coated 6-well dishes, irradiated, treated with TH588, and incubated for 10 d. The number of plated cells was adjusted experimentally for each cell line and treatment to avoid confluence or a complete wipe out. After fixation in 0.25% paraformaldehyde (PFA), cells were washed with 70% ethanol and stained with 1 g/L Coomassie Brilliant Blue for 1 h. Colonies of a least 50 cells were counted. Plating efficiency (PE) was calculated by dividing the number of colonies by the number of plated control cells. To compare different treatments, survival fractions (SF) were calculated as $SF=\frac{colonies}{plated\kern0.5em cells\kern0.5em x\kern0.5em PE}$

### Apoptosis quantification

Apoptosis was quantified by measurement of caspase-3 activity in whole cell extracts as described previously [18]. For each sample 50 μg of protein was incubated with 50 μM Ac-DEVD-AMC in caspase-3 substrate buffer at 37 °C for 4 h. The fluorescent product 7-amino-4-methylcoumarin (AMC) was quantified every 10 min (excitation 360 nm, emission 460 nm) in a fluorescence reader (Synergy HT, BioTek, Bad Friedrichshall, Germany). Replicate values (*n*  =  3 per experiment) of a single timepoint in the linear range of the reaction were plotted.

### Western blotting

Whole cell extracts were prepared in RIPA lysis buffer and processed for Western blotting as published previously [16]. Proteins were separated on 10% reducing SDS gels and blotted onto PVDF membranes. Unspecific binding sites were blocked with 5% skimmed milk in TBST. Rabbit polyclonal antibodies against MTH1 (Novus Biologicals, Littleton, CO, USA) and HIF-1β (Cell Signaling, Cambridge, UK) as well as monoclonal mouse antibodies against β-actin (Sigma), were used overnight at 4 °C. HRP-conjugated secondary antibodies (Dako, Hamburg, Germany) were used for 1 h at room temperature.

### Statistical analysis

All results were confirmed in at least two further independent experiments. Data presented in bar graphs include at least three independent samples. Bars represent mean plus standard deviation. When two groups were compared Student’s *t*-test was applied. For multiple group comparison two-way ANOVA followed by Bonferroni or Dunnett’s post-hoc test were used. In all figures * indicates *p* < 0.05, while ** indicates *p* < 0.01 and *** means *p* < 0.001.

## Results

### TH588 treatment reduces cell viability in vitro

**Fig. 1:**
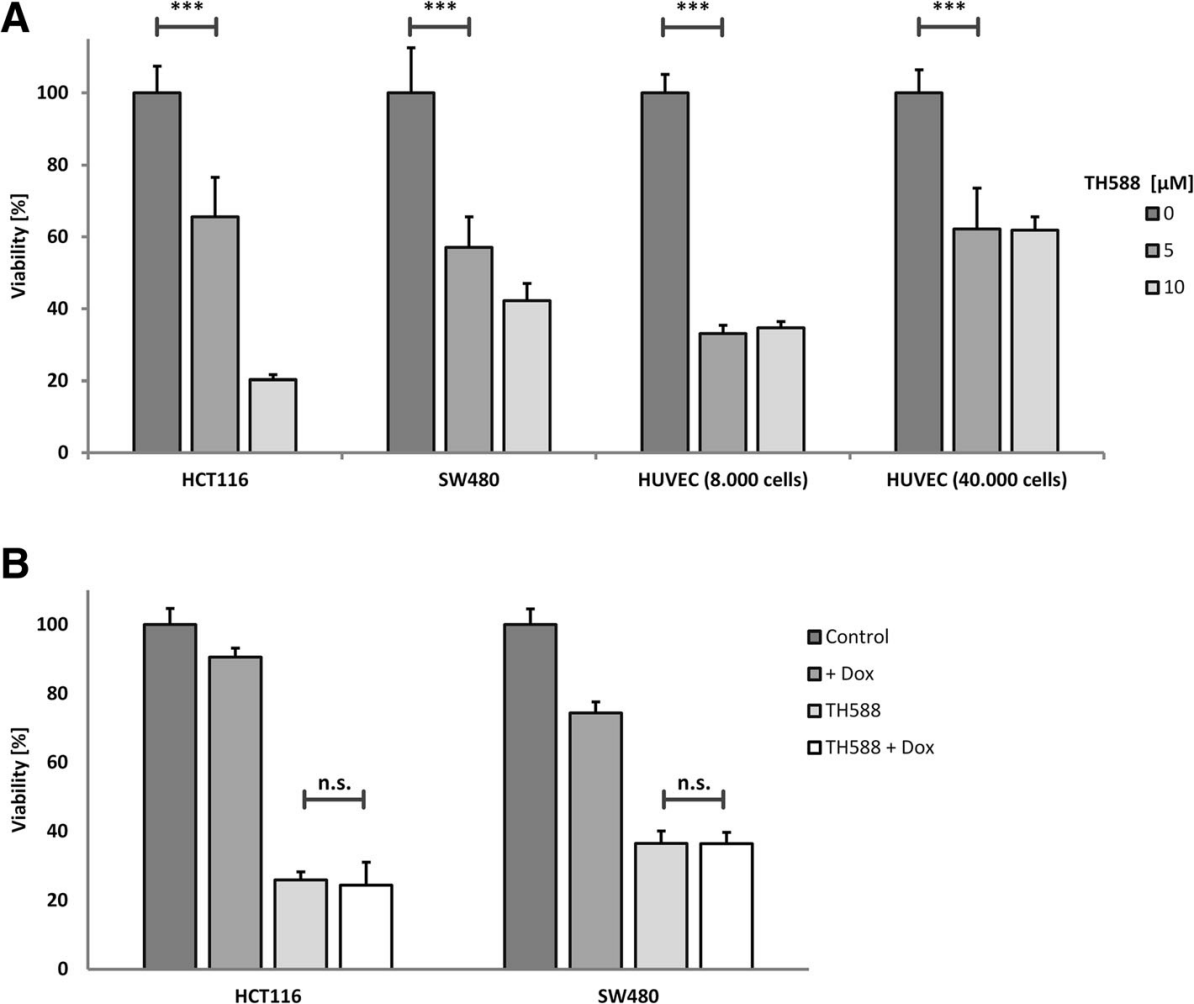
Dose-dependent decrease of viability in cancer and non-cancer cell lines after TH588 treatment. HCT116, SW480 and HUVEC were plated on 96-well-plates. Incubations took place for 72 h. MTT reagent was added 4 h before lysis of the cells. Viability was assessed by means of an MTT assay. Values are given as % of the untreated control in each group. Bonferroni’s post-hoc test was used to determine significant differences between groups. **a** Viability of different cell lines after TH588-treatment. HUVEC were in a proliferating state when 8.000 cells per well had been plated. 40.000 HUVEC per well led to a confluent cell layer. Cells were treated with final concentrations of 5 or 10 μM TH588 (**b**) Viability of cells treated with 5 μM TH588, doxycycline for knockdown induction, or both

**Fig. 2:**
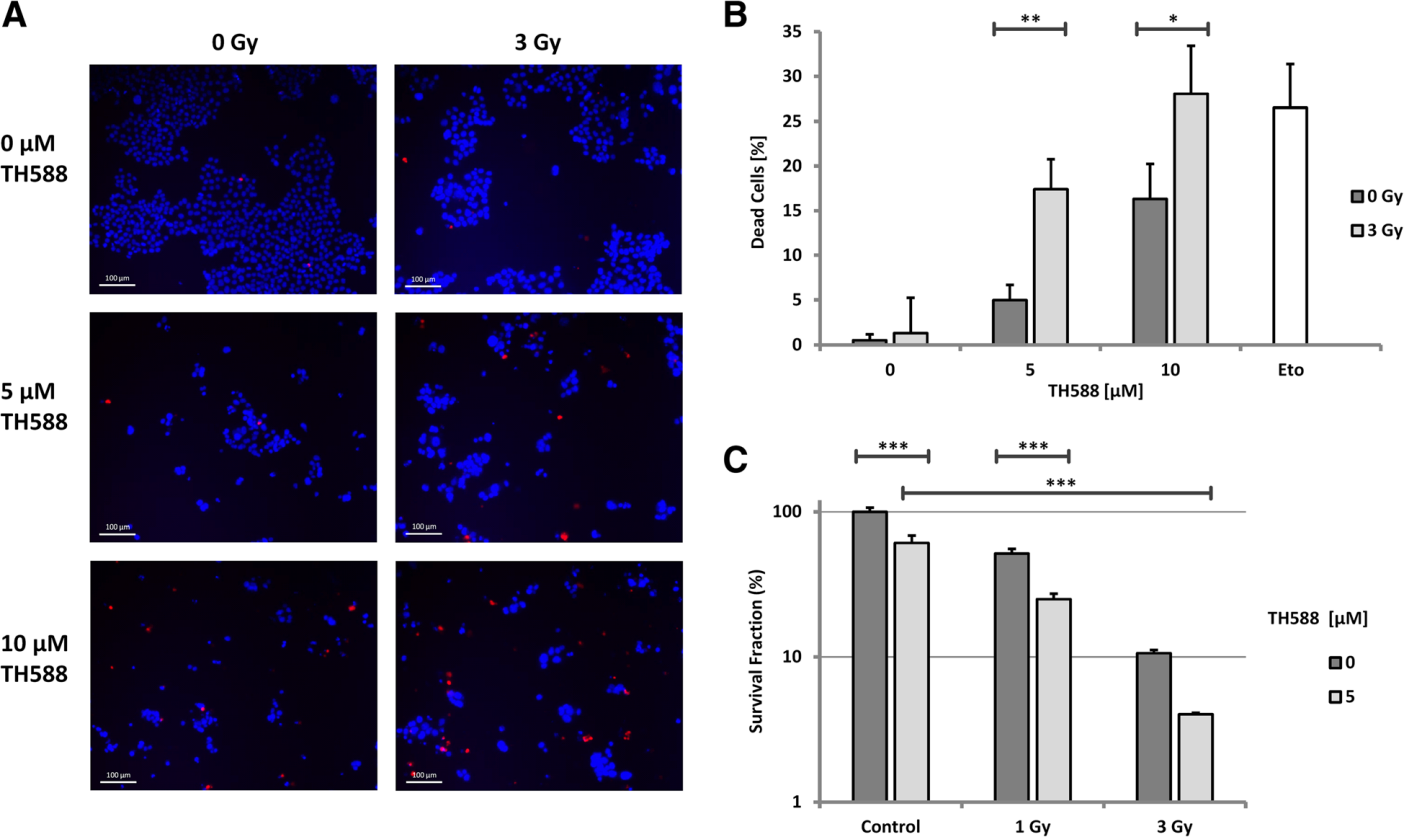
TH588 and IR induce cell death in HCT116. For all experiments with chemical agents control cells were treated with vehicle only (DMSO). Cells were irradiated and immediately treated afterwards and then incubated for 48 h. **a** After staining with PI (red) and Hoechst 33342 (blue) immunofluorescence images were recorded**. b** Dead cells and total cells were counted. The ratio of dead divided by total cells is displayed**.** Etoposide was used as a positive control. Bonferroni’s test was used as post-hoc test. **c** Colony formation assays were performed in collagen coated dishes. After irradiation cells were treated with TH588 and incubated for 10 days. Only colonies with more than 50 cells were counted. Bonferroni’s post-hoc test was used to determine significant differences between groups

To investigate whether treatment with the MTH1 inhibitor TH588 affects cell viability in vitro we treated HCT116, SW480 and HUVEC with increasing concentrations of the compound for 3 days. Viability was significantly reduced in all cell lines with a similar dose response after TH588 treatment as determined by means of an MTT assay (Fig. 1a). In HCT116 10 μM TH588 was strikingly more effective than 5 μM while in SW480 the 2 concentrations were similarly effective. Additional knockdown of MTH1 after 5 μM inhibitor treatment did not further enhance this impairment (Fig. 1b). Gad et al. proposed MTH1 to be non-essential in normal cells by reporting a considerably less toxic effect of TH588-treatment on primary and SV40- or hTERT-transformed fibroblasts [6]. Because HUVEC are widely used as a model system for untransformed human cells, we tested their response to MTH1 inhibition. We used HUVEC at low density, i.e. in a highly proliferating state as well as at high confluence which was supposed to resemble a non-proliferating situation. After plating 8.000 cells viability was significantly reduced to 40% of the control at a concentration of 5 μM TH588 (Fig. 1a) and thus very similar to the cancer cell lines tested. When 40.000 cells per well were plated the reduction of viability was less pronounced but still significant. A reduction of viability as demonstrated by MTT assays is not necessarily caused by cell death induction but may also allude to a reduction of proliferation. Therefore, we assessed cell survival more directly by propidium iodide (PI) staining and colony formation assays. HCT116 were stained with propidium iodide (PI) which selectively stains dead cells red and with the DNA dye Hoechst 33342 which stains all nuclear DNA blue. In HCT116, total cell death as determined by calculating the ratio of dead to vital cells was significantly increased by treatment with TH588 (Fig. 2a and b). In line with the MTT assays 10 μM were considerably more efficient than 5 μM. Irradiation with 3 Gy which is a rather low dose in HCT116 alone did not have a significant effect which was, however, strikingly increased when IR was combined with TH588 treatment. The most significant effect was caused by combination of 3 Gy and 10 μM TH588 (Fig. 2b) which was as detrimental to the cells as the established cytotoxic substance etoposide. Longer term effects of TH588 and IR on survival and growth of HCT116 were tested by colony formation assay. These experiments demonstrated a significant reduction of long term survival after treatment with 5 μM of TH588 alone or in combination with an irradiation dose of 1 or 3 Gy (Fig. 2c). Treatment with 5 μM TH588 plus 1 Gy reduced the survival fraction (SF) by 75% while 5 μM TH588 or 1 Gy alone only led to a reduction of 40 or 50%, respectively. The most potent reduction of the survival fraction was measured after co-treatment with 3 Gy and 5 μM of TH588 when 95% less actively dividing cells were observed as compared to the control experiment.

### Hypoxic cells are susceptible to TH588 treatment

**Fig. 3:**
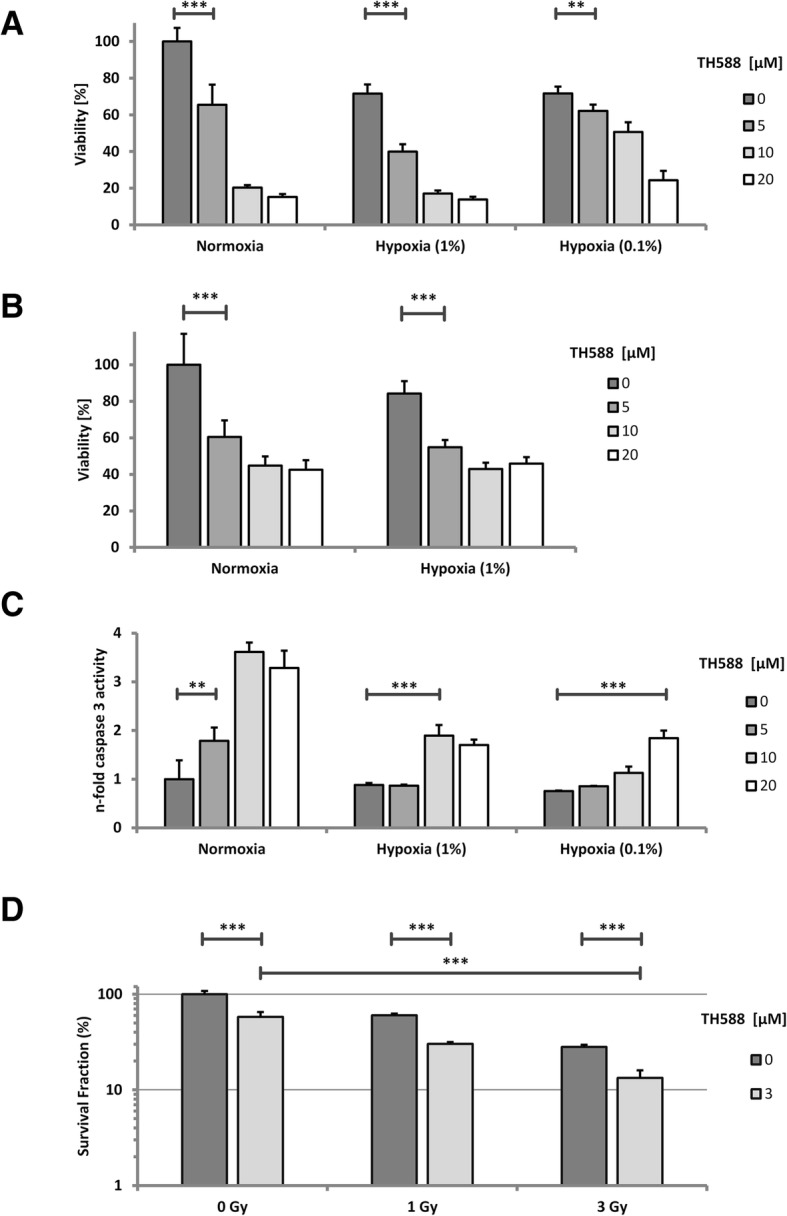
Hypoxic HCT116 cells are susceptible to TH588 treatment. Hypoxic samples were placed in a hypoxia workstation for 12 h before treatment to adapt the cells to low oxygen concentrations. TH588 was applied in the hypoxic chamber. HCT116 (**a**) and SW480 (**b**) cells were treated with TH588 in hypoxia or normoxia and incubated for 72 h. The MTT reagent was added 4 h before lysis. Graphs were normalized to the untreated and un-irradiated control. Bonferroni’s test was used as post-hoc test. **c** Apoptosis induction was analyzed by caspase-3 assay. The cells were treated as in A. Whole cell protein extracts were produced after 72 h. Dunnett’s test was used as post-hoc test. **d** Colony formation assay of cells cultured at 1% oxygen. Immediately after irradiation, the cells were treated with TH588 and incubated for 10 days. Bonferroni’s post-hoc test was used to determine significant differences between groups

Hypoxia is known to adversely affect the efficacy of IR and chemotherapy. Therefore we tested whether TH588 also interferes with viability, apoptosis induction and colony formation of hypoxic cells. Moderate hypoxia (1% O_2_) reduced viability by approximately 40%, severe hypoxia had virtually the same effect (Fig. 3a). 5 μM TH588 reduced viability in HCT116 cells under normoxic cell culture conditions by almost 40%, higher doses were more efficient (Fig. 3a). In moderate hypoxia, TH588 substantially reduced viability with a similar dose response as compared to normoxia. Remarkably, in severe hypoxia higher doses of TH588 we required to give the same effect. We have also assessed viability of SW480 incubated in hypoxia with different concentrations of TH588. Low dose treatment led to a very similar response as compared to HCT116, while these cells were slightly less sensitive to higher doses of TH588 (Fig. 3b). To test whether loss of viability was caused by induction of apoptosis we used caspase-3 activity assays. Indeed, caspase-3 activation was demonstrated in normoxia and in hypoxia after application of TH588 while hypoxia alone did not cause apoptosis (Fig. 3c). Under normoxic conditions, a significant increase was measured after application of 5 μM TH588, maximal induction (3.5fold) was achieved with 10 μM. Again, in hypoxia higher concentrations of TH588 were required for induction of apoptosis. Interestingly, in normoxia 5 μM TH588 was the maximal pro-apoptotic concentration while it was 10 μM in moderate hypoxia (1% O_2_) and 20 μM in severe hypoxia (0.1% O_2_). In general, induction of apoptosis by TH588 was less pronounced in hypoxia as compared to normoxia.

### Radiation sensitivity of hypoxic cells is increased by TH588 treatment

We next tested whether hypoxic cells are sensitized to IR by TH588. For this experiment all cells were incubated in moderate hypoxia, i.e. 1% O_2_ (Fig. 3d). Colony formation of HCT116 was significantly reduced to 60% of the untreated, unirradiated control after treatment with 3 μM TH588 which is very similar to the reduction observed after an irradiation dose of 1 Gy without TH588 treatment. Remarkably, at all irradiation doses 3 μM TH588 reduced the number of colonies formed to an almost identical proportion from 60 to 30% after 1 Gy and from 28 to 12% after 3 Gy strongly indicating that TH588-treatment acted additively to IR in hypoxia.

### Cell line specific tumor cell death after knockdown of MTH1

**Fig. 4:**
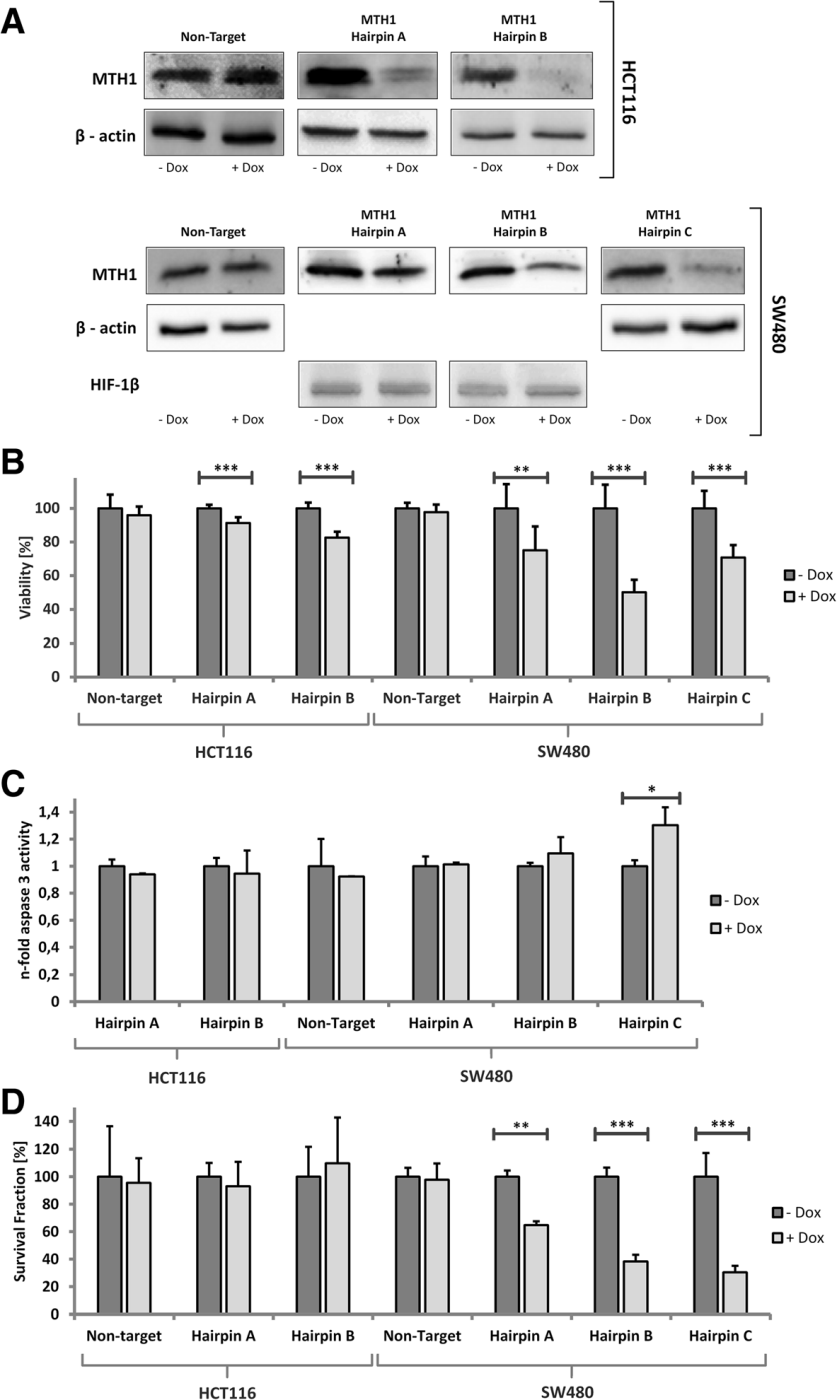
Survival of SW480 cells, but not of HCT116 cells depends on MTH1. **a** Knockdown efficiency was demonstrated by Western blotting. HCT116 and SW480 cells were harvested 72 h after knockdown induction. HIF-1β and β-actin served as control genes to confirm equal loading and protein transfer. **b** MTT assay in response to lentiviral knockdown of MTH1. Viability was measured 72 h after knockdown induction. **c** Apoptosis induction was assessed by measurement of caspase-3 activity 72 h after knockdown induction. **d** To calculate clonogenic survival, colonies were counted 10 days after knockdown induction. Bars were normalized to the control in each group. For statistical analysis Student’s t-test was used

Because it is highly controversial at present whether the effects of TH588 result from MTH1 inhibition only, we attempted to reproduce the inhibitor result with a doxycyclin-inducible lentiviral knockdown of MTH1 in in the two commonly used colon tumor cell lines HCT116 and SW480. We used two different hairpin sequences (*hairpin A and B*) which efficiently reduced protein expression by 70–90% in HCT116 as demonstrated by Western blotting (Fig. 4a). Hairpin A was less efficient in SW480. In contrast to TH588 treatment, the effect on viability was weak in HCT116 although it reached statistical significance while all shRNAs reduced viability significantly in SW480 (Fig. 4b). Apoptosis induction was not detectable in HCT116 cells (Fig. 4c). In SW480, the hairpins A and B did not have a significant effect on caspase activity while a third hairpin sequence also targeting MTH1 (hairpin C), which had been used by another group earlier [6], significantly induced apoptosis (Fig. 4c). Colony formation assays performed with the hairpin sequences A and B led to clearly discrepant results when we compared SW480 and HCT116 cells: while we readily demonstrated reduced colony formation in SW480, both hairpins failed to affect survival in HCT116 (Fig. 4d). In addition to our own lentiviral transductions, Dr. Helleday also generously provided us with the SW480 subclone which had been used in the original publication [6]. We observed that the survival fraction was reduced substantially after induction of hairpin expression which is in line with the results published previously.

## Discussion

The enzyme MTH1 has attracted substantial attention in biomedical research since it was proposed to be a “druggable” target in cancer therapy [6]. This was based on the observation that cancer cells display an elevated level of reactive oxygen species (ROS) [19, 20] along with permanently activated defense mechanisms as expertly reviewed recently [21]. Specifically to prevent oxidative DNA damage, elaborate repair mechanisms have evolved [22]. One of these is MTH1 function which prevents incorporation of oxidatively damaged nucleotides in replicating DNA. Therefore it was assumed that when MTH1 is inhibited, permanently high levels of ROS should lead to lethal DNA damage in cancer cells. Particularly prone to oxidative damage is guanine which gives rise to 8oxoG in DNA and transversion mutations upon the next replication cycle. Indeed, MTH1 was observed to be the only enzyme with significant 8-oxo-dGTP hydrolyzing activity [23] and MTH1-deficient mice develop tumors of lung, liver and stomach spontaneously [24]. However, soon after the proposal of MTH1 as a target, a number of groups reported that MTH1 inhibition either led to MTH1 independent cytotoxic effects or failed to affect cell viability in cell culture [13, 14] and in mice [25].

In this situation it was our aim to investigate whether the well-characterized MTH1 inhibitor TH588 has detrimental effects on cultured colorectal carcinoma cells and whether these effects are attributable to MTH1 inhibition. We were particularly interested to elucidate whether TH588 affects cell viability in situations which interfere with oxidative stress such as IR and hypoxia. This issue seemed of vast relevance since radiation exerts its effect largely by generation of ROS. Hypoxia is well known to negatively affect cellular radiation sensitivity. From the proposed mechanism of action it seemed conceivable that TH588 acts as a radiosensitizer in hypoxia which is an aspect of paramount importance that has been neglected so far.

Indeed, our experiments with TH588 consistently demonstrated that severe effects on cell viability are detectable in normoxia as well as in moderate and severe hypoxia in short term cell survival assays and in CFAs. Remarkably, these effects were significant in all cell lines tested, i.e. in both carcinoma cell lines and in HUVEC although HUVEC were only tested in MTT assays. Notably, proliferating and confluent endothelial cells were affected by TH588. Reduction of endothelial cell growth is not necessarily a drawback for a substance designated to be a cancer therapeutic since endothelial cell proliferation occurs very rarely in the adult organism apart from tumor angiogenesis. Altogether, however, since the effects on HUVEC were significant, we regard the statement that TH588 does not affect untransformed cells in general as premature.

With respect to hypoxia it is still controversial whether hypoxia affects generation of ROS at all and, if so, whether ROS are increased [26–28] or decreased in hypoxia [29, 30]. In the setting of irradiation, decreased toxicity in hypoxia is obviously well in line with low substrate O_2_ levels and thus reduced ROS generation. In our experiments, the contribution of TH588 to cytotoxicity when applied in combination with IR was very similar in normoxia and in moderate hypoxia. Reaching the same amount of toxicity in severe hypoxia required moderately higher TH588 concentrations. These results demonstrate that TH588 is also efficient in hypoxia and can add to IR toxicity in hypoxic areas of the tumor which are known to be resistant to IR otherwise.

As mentioned above, independent groups have confirmed cytotoxic effects of TH588 in several studies [7, 13]. Some studies have also identified MTH1 inhibitors with similar or even lower IC50 [13, 25] which either caused cytotoxic effects regarded as MTH1 independent or had no significant impact on cell viability thus challenging the concept of MTH1 inhibition as an anti-tumor strategy. We used inducible lentiviral inhibition of MTH1 expression as an alternative route to inhibition of the enzyme. Three independent shRNA sequences were shown to efficiently downregulate enzyme expression in HCT116 and SW480 cells. While reduction of survival as determined by CFA in SW480 was significant in response to all shRNAs, long term survival remained unchanged in HCT116. These results strongly support the conclusion that in SW480 MTH1 inhibition is causal for the detrimental effects which we have observed because it is highly unlikely that three unrelated shRNA sequences lead to the same cellular phenotype. On the other hand, we demonstrate that inhibition of MTH1 expression has no discernible effect on HCT116 which does indicate that the TH588 effect which was easily detectable is unrelated to expression or function of MTH1. Interestingly, it was reported very recently that tubulin was the primary target of TH588 [14] although it was not formally excluded that MTH1 interacts with tubulin. Nevertheless, since TH588 caused cellular damage while shMTH1 did not, it is likely that TH588 affects HCT116 in an MTH1-independent manner.

## Conclusions

Collectively, our data demonstrate that the compound TH588 which has been developed as an inhibitor of the 8-oxo-GTP hydrolyzing enzyme MTH1 impairs survival of cultured colon cancer cells and reduces viability of proliferating endothelial cells. TH588 has properties of a radiosensitizing compound and, most strikingly, also increases the efficiency of IR on hypoxic tumor cells. According to our data, the cellular responses to TH588 are determined by MTH1-dependent and MTH1-independent effects. Presence and cellular importance of TH588 target structures are likely to determine sensitivity in a cell type specific manner. It should be clarified in further experiments which cell lines and tumor entities are sensitive to treatment with this promising compound.

## Abbreviations

AMC: 7-amino-4-methylcoumarin
CFA: Colony formation assay
DMSO: Dimethylsulfoxide
DOX: Doxycycline
HUVEC: Human umbilical vein endothelial cells
IC50: Half-maximal inhibitory concentration
IR: Ionizing radiation
MTH1: MutT homologue-1
MTT: 3-(4,5-dimethylthiazol-2-yl)-2,5-diphenyltetrazolium bromide
PE: Plating efficiency
PFA: Paraformaldehyde
PI: Propidium iodide
ROS: Reactive oxygen species
SF: Survival fraction
shRNA: Short hairpin RNA
